# Global, regional, and national burdens and trends of migraine among males aged 10–59 years from 1990 to 2021: insights from the Global Burden of Disease study 2021

**DOI:** 10.3389/fneur.2025.1585512

**Published:** 2025-06-06

**Authors:** Haonan Zhao, Qingfang Wang, Zhang Chen, Wenxia Zhu

**Affiliations:** ^1^Department of Neurology, Shanghai East Hospital, Tongji University School of Medicine, Shanghai, China; ^2^Department of Cardiothoracic Surgery, Shanghai East Hospital, Tongji University School of Medicine, Shanghai, China

**Keywords:** migraine, disease burden, GBD 2021, epidemiology, sex differences

## Abstract

**Background:**

Migraine, a prevalent neurological disorder, significantly impacts quality of life across populations. Although various demographic groups are impacted, existing research has focused predominantly on the general population, women, and adolescents, with insufficient emphasis on the burden experienced by men. This study aims to analyze data from the Global Burden of Disease (GBD) study spanning from 1990 to 2021, with the objective of elucidating the global prevalence, incidence, and disability-adjusted life years (DALYs) associated with migraine in males aged 10–59 years. Our investigation seeks to provide insights into this underexplored aspect of migraine’s impact over three decades. Epidemiological Transition refers to a shift in the burden of disease from communicable diseases to chronic non-communicable diseases, which in this study is reflected in the rising relative burden of migraine among men. Secondary Prevention (Secondary Prevention) reduces the frequency of migraine attacks through early diagnosis and intervention, e.g., use of prophylactic medications; Tertiary Prevention (Tertiary Prevention): reduces the degree of disability in diagnosed patients, e.g., cognitive-behavioral therapy.

**Results:**

From 1990 to 2021, there was a significant increase in the burden of migraine among males aged 10–59 years worldwide. The number of incident cases rose by 46.55%, the number of prevalent cases rose by 56.54%, and the number of DALYs increased by 56.95%. The middle-SDI region showed the fastest burden growth. At the country level, Belgium had the highest prevalence and DALYs, whereas Indonesia and the Philippines had the highest incidence. Age-period-cohort analysis revealed a peak incidence in the 10–14 years age group, with the prevalence and DALYs peaking in the 30–44 years age range. Population growth was the primary driver of increased burdens in most regions. Projections suggest a continued increase in migraine burden among this population in the future.

**Conclusion:**

This paper is the first to systematically analyze the age-period-cohort effect of migraine in men and to reveal a unique upward trend in the burden of migraine among men in the high-income Asia-Pacific region (in contrast to the trend among women). From 1990 to 2021, the migraine burden among males aged 10–59 years has generally increased and is expected to continue rising. Notably, the incidence is highest among adolescents (10–14 years), whereas the prevalence and DALYs peak in the middle-aged group (30–44 years). To address this, we should focus on primary prevention in adolescents and implement secondary and tertiary prevention strategies for the middle-aged population to reduce the overall migraine burden in males. Additionally, in the high-income Asia–Pacific region, the increasing trend in migraine burden differs from previous research findings.

## Introduction

Migraine is a neurological disorder characterized by recurrent pulsating headaches, typically unilateral, with moderate to severe intensity, and is accompanied by neurological symptoms such as nausea and vomiting (1). It consistently contributes significantly to the global disease burden, making it a major public health issue worldwide (2). Although migraine poses a significant burden across all age groups, ranking third among neurological disorders in terms of overall disease burden, its impact varies considerably by age. Among adolescents (10–19 years), migraine is the leading cause of neurological disease burden. For adults aged 20–59 years, migraine ranks as the second most burdensome neurological condition (3). These age groups are critical for education, career development, and social relationships (4–7). Furthermore, the ICHD-3 diagnostic criteria explicitly highlight the differences between diagnostic standards for young children and adults (8). Existing studies demonstrate that due to poor cooperation and inability to articulate symptoms clearly in this age group, the clinical misdiagnosis rate remains high (9, 10). Moreover, World Health Organization’s previous age stratification criteria defined 60 years as the threshold for the elderly population, while the high prevalence of comorbidities such as hypertension and cerebrovascular diseases in older adults can confound migraine diagnosis (11). Based on these considerations, we ultimately selected individuals aged 10–59 years as our target population. Numerous studies have demonstrated significant sex differences in migraine (12–14), leading past research to focus primarily on women or the general population, with insufficient attention given to young and middle-aged men. Research predicting the future burden of migraine indicates that while the burden for women will show a slight downward trend in the coming decades, it will increase for men (15). Therefore, understanding the epidemiology of migraines in males aged 10–59 years allows us to implement early detection, prevention, and management strategies to reduce the long-term burden and improve outcomes for this group. Moreover, it could alleviate the overall migraine burden across all populations globally. This study focused on the male population aged 10–59 years based on the following considerations: first, this age group has been underrepresented in previous migraine studies, and the burden of disease in adolescent males in particular (aged 10–19 years) is often underestimated; and second, 59 years of age as an upper limit excludes co-morbid interferences of neurodegenerative disorders that are more highly correlated with age (e.g., Parkinson’s disease), while preserving the role of migraine as the typical feature of primary headache. Although migraine is still present in the elderly, this study aims to provide an in-depth analysis of the specific burden patterns in working-age and adolescent males.

## Method

### Data sources and disease definitions

The data utilized in this study were sourced from the Global Health Data Exchange’s GBD Results Tool.^1^ This resource offers comprehensive details on the data, methodologies, and statistical models used in prior reports (16, 17). According to the GBD 2021, migraine is recognized as a disabling primary headache disorder characterized by recurrent, moderate to severe, pulsating pain typically located on one side of the head. This study does not distinguish between migraines with or without aura, as most epidemiological research generally addresses migraines. A diagnosis is made if a patient’s symptoms align with all five major diagnostic criteria outlined by the International Classification of Headache Disorders, 3rd edition (ICHD-3). In the International Classification of Diseases (ICD), 9th and 10th editions, migraines are coded as 346–346.93 and G43-G43.919, respectively (16). Urbanization and work stress explained part of the trend (e.g., East Asia OR = 1.32, 95% CI 1.21–1.44), but the decline in incidence (APC = −1.2%) in high-income countries (e.g., Scandinavia) may be associated with a higher diagnostic threshold (diagnostic shift from headache to chronic daily headache) and a cultural shift in pain management. Future validation of differences in reporting rates in conjunction with medical records is needed.

### Statistical analysis

This investigation leverages data from the Global Burden of Disease study 2021 (GBD 2021) to conduct a comprehensive epidemiological analysis of migraine among males aged 10–59 years. This study examines trends at the global, regional, and national scales, encompassing worldwide patterns, Socio-demographic Index (SDI) regions, 21 GBD regions, and 204 individual countries over the period from 1990 to 2021.

To identify significant shifts in trends, we employed joinpoint regression modeling (18). Additionally, we utilized age-period-cohort models to analyze migraine, which is particularly valuable for its capacity to disentangle the influences of age, period, and cohort effects on health outcomes, thereby offering a nuanced perspective on trend dynamics (19). To elucidate the underlying factors driving changes in disease burden, we implemented a decomposition analysis, dissecting the variations into three key components: demographic aging, population expansion, and epidemiological shifts (20). Our forward-looking analysis incorporated the nordpred model and Bayesian Age-Period-Cohort (BAPC) methodology to project future migraine trends within the target population (21, 22). The BAPC model assumes a linear change in incidence over time, whereas the Nordpred model uses an age-period-cohort design. We verified the model stability through goodness-of-fit tests (e.g., R (2)=0.85, *p* < 0.001) for historical data and acknowledged that the predicted results are highly sensitive to future social changes such as healthcare accessibility and rate of urbanization, and therefore the results should be considered as trend-referenced rather than precise predictions.

The joinpoint analyses were executed via joinpoint regression software version 4.9, which was developed by the Statistical Research and Applications Branch of the National Cancer Institute, United States. For the construction of age–period–cohort models, we relied on Stata 14.0 software (StataCorp LP, TX, United States). All other statistical procedures were performed via R software, version 4.3.3.

## Result

### Global trends and regional disparities in migraine burden

**Figure 1:**
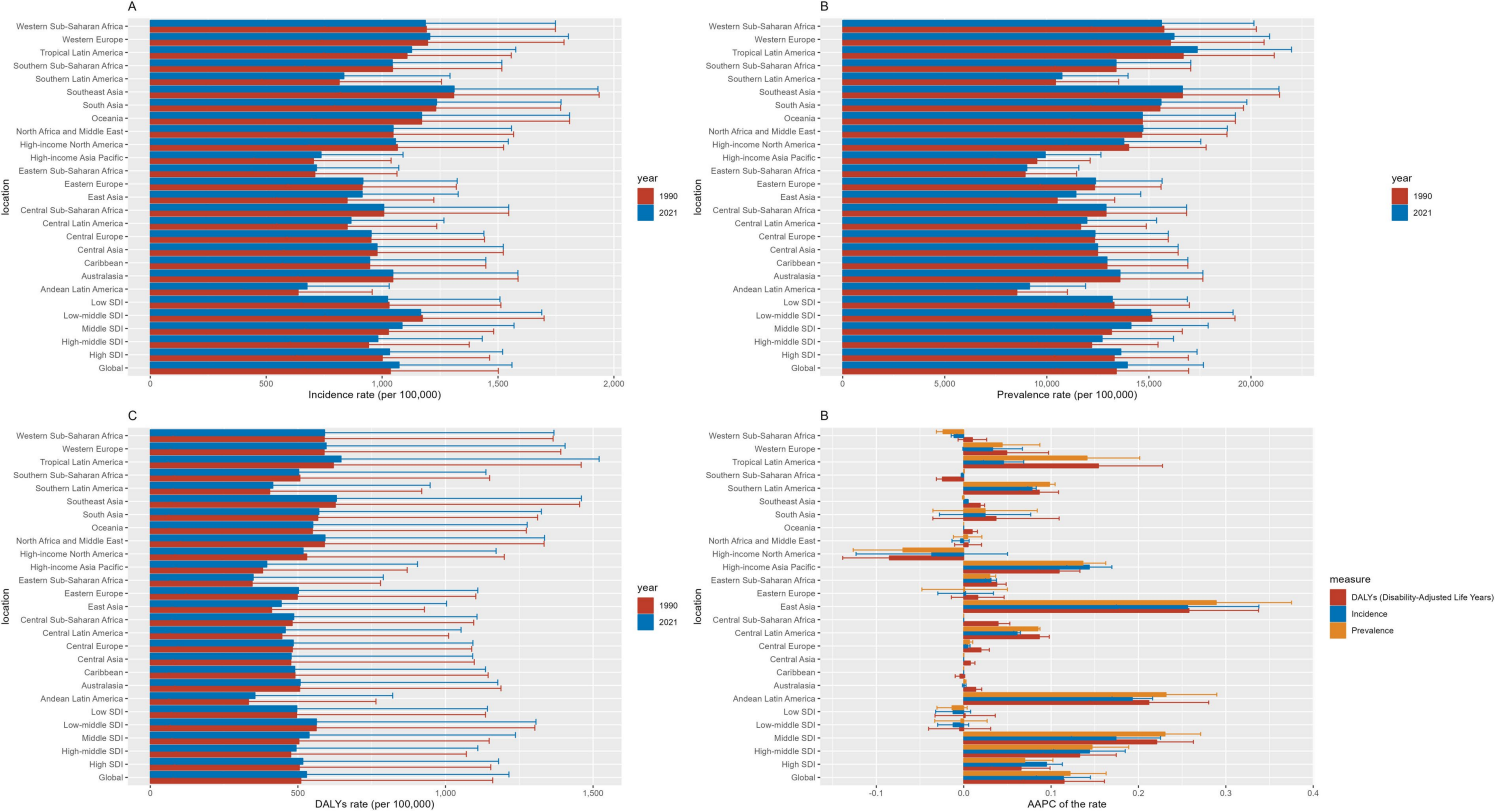
Incidence, prevalence, and DALYs of migraine among 10–59 males in 1990 and 2021. **(A)** Incidence, **(B)** Prevalence, **(C)** DALYs, **(D)** the AAPC of incidence, prevalence and DALYs.

Our analysis of migraine burden among males aged 10–59 years revealed significant global increases in prevalence, incidence, and disability-adjusted life years (DALYs) from 1990 to 2021 (Table 1; Figure 1; Supplementary Tables S1, S2). Globally, incident cases rose by 46.55% (19878800.47 [95% UI: 12810023.19–28749405.83] to 29132612.28 [18656276.71–42425323.21]), prevalent cases by 56.54% (246281388.64 [191897520.31–311672412.06] to 385501430.49 [301664369.52–488363158.34]), and DALYs by 56.95% (9335098.86 [1340152.77–21382278.40] to 14653782.98 [2140199.99–33543835.61]). Annual average percentage changes (AAPCs) for incidence (0.11, 95% CI: 0.08–0.14), prevalence (0.12, 0.08–0.16), and DALYs (0.11, 0.07–0.16) confirmed sustained upward trends.

**Table 1:** Numbers and ASR per 100,000 cases of incidence of migraine among 10–59 males in 1990 and 2021, along with AAPC in ASR per 100,000 cases from 1990 to 2021, categorized by global, SDI, and GBD regions.

Characteristic	Number in 1990 (95% UI)	Age-standardized rate in 1990 (95% UI)	Number in 2021 (95% UI)	Age-standardized rate in 2021 (95% UI)	AAPC (95% CI)
Global	19878800.47 (12810023.19 to 28749405.83)	1035.7 (665.56 to 1501.28)	29132612.28 (18656276.71 to 42425323.21)	1073.26 (688.66 to 1560.21)	0.11 (0.08 to 0.14)
High SDI	3001533.77 (1883834.68 to 4417117.21)	1000.95 (633.37 to 1463.75)	3377424.31 (2118565.27 to 5024201.48)	1032.27 (656.68 to 1,520)	0.09 (0.08 to 0.11)
High-middle SDI	3,611,589 (2309608.82 to 5291606.21)	941.69 (604.21 to 1375.83)	4144468.32 (2628053.6 to 6092900.82)	981.9 (628.14 to 1432.21)	0.14 (0.1 to 0.18)
Middle SDI	6651376.24 (4317711.75 to 9566220.69)	1027.34 (664.68 to 1481.32)	9249762.01 (5950378.39 to 13414101.19)	1086.25 (701.93 to 1569.13)	0.17 (0.12 to 0.23)
Low-middle SDI	4859111.95 (3155514.6 to 6,975,089)	1173.73 (754.45 to 1698.82)	8228558.71 (5,296,222 to 11887039.52)	1166.28 (748.76 to 1688.65)	−0.01 (−0.03 to 0.01)
Low SDI	1737310.92 (1115252.37 to 2519396.61)	1,030 (651.04 to 1512.44)	4110633.28 (2617698.32 to 5983589.55)	1023.78 (643.11 to 1508.39)	−0.01 (−0.03 to 0.01)
Andean Latin America	88140.32 (55371.45 to 130478.18)	637.77 (392.97 to 957.26)	159031.43 (95290.75 to 242490.76)	676.4 (406.33 to 1030.61)	0.19 (0.17 to 0.22)
Australasia	73419.49 (44315.05 to 111950.25)	1046.24 (635.74 to 1586.45)	98827.96 (59540.06 to 150905.22)	1046.44 (636.41 to 1585.81)	0 (0 to 0)
Caribbean	118571.37 (71733.02 to 180615.96)	946.47 (570.34 to 1447.43)	150582.46 (90021.82 to 231388.72)	946.47 (570.34 to 1447.43)	0 (0 to 0)
Central Asia	233046.39 (138133.11 to 361295.09)	978.46 (576.3 to 1522.86)	323682.09 (190610.31 to 504745.83)	978.46 (576.3 to 1522.86)	0 (0 to 0)
Central Europe	408080.61 (252254.15 to 619597.32)	952.11 (589.55 to 1441.76)	331668.96 (203572.29 to 504824.07)	953.14 (592.23 to 1439.13)	0 (0 to 0.01)
Central Latin America	512283.24 (334399.98 to 735591.8)	849.7 (545.35 to 1235.79)	756830.64 (483087.8 to 1109079.9)	865.98 (554.61 to 1266.76)	0.06 (0.06 to 0.06)
Central Sub-Saharan Africa	185468.54 (111196.29 to 280572.2)	1007.28 (594.65 to 1546.21)	494786.35 (296390.45 to 749153.85)	1007.28 (594.65 to 1546.21)	0 (0 to 0)
East Asia	3994843.01 (2557962.69 to 5764110.93)	849.1 (545.43 to 1223.15)	4411314.48 (2823219.02 to 6445798.27)	914.2 (589.14 to 1328.23)	0.26 (0.17 to 0.34)
Eastern Europe	677591.61 (439058.45 to 982744.55)	914.26 (594.83 to 1320.09)	571086.72 (369789.93 to 830370.15)	917.36 (597.37 to 1,324)	0 (−0.03 to 0.03)
Eastern Sub-Saharan Africa	447854.68 (281081.02 to 660704.44)	709.9 (437.87 to 1064.25)	1094910.91 (681466.47 to 1617695.27)	716.97 (441.24 to 1071.98)	0.03 (0.03 to 0.04)
High-income Asia Pacific	429011.81 (270977.29 to 636293.91)	703.9 (447.4 to 1038.79)	363585.29 (226213.31 to 543346.64)	736.84 (464.8 to 1090.3)	0.14 (0.12 to 0.17)
High-income North America	1010137.32 (644886.75 to 1449826.39)	1066.07 (684.01 to 1524.04)	1174423.95 (749426.46 to 1722483.31)	1057.92 (679.72 to 1544.3)	−0.04 (−0.12 to 0.05)
North Africa and Middle East	1311050.82 (823420.74 to 1933831)	1047.3 (646.33 to 1567.35)	2446628.73 (1512802.76 to 3642343.56)	1047.74 (647.52 to 1558.06)	0 (−0.01 to 0.01)
Oceania	28280.55 (17063.48 to 43237)	1170.48 (695.6 to 1808.68)	59683.64 (35751.49 to 91696.7)	1170.48 (695.6 to 1808.68)	0 (0 to 0)
South Asia	4913585.85 (3201588.03 to 7026390.05)	1231.39 (796.83 to 1770.07)	8601780.9 (5572028.56 to 12336086.89)	1,235 (799.62 to 1771.95)	0.02 (−0.03 to 0.08)
Southeast Asia	2219637.96 (1400864.21 to 3260787.99)	1308.88 (819.57 to 1936.25)	3273693.25 (2048976.76 to 4836140.93)	1310.8 (823.11 to 1931.14)	0 (0 to 0.01)
Southern Latin America	137795.21 (81623.95 to 211655.33)	815.22 (481.49 to 1256.03)	189316.91 (111609.3 to 294522.94)	835.14 (495.84 to 1292.93)	0.08 (0.07 to 0.08)
Southern Sub-Saharan Africa	191393.92 (122909.18 to 275627.75)	1044.94 (666.01 to 1516.5)	303274.26 (193365.93 to 440042.08)	1044.24 (664.77 to 1516.43)	0 (0 to 0)
Tropical Latin America	637998.18 (436975.9 to 888177.7)	1107.52 (747.66 to 1557.16)	836342.75 (561485.08 to 1181198.03)	1127.39 (766.99 to 1576.57)	0.05 (0.02 to 0.07)
Western Europe	1497073.62 (915689.41 to 2253262.85)	1196.31 (741.46 to 1784.79)	1486756.82 (908601.88 to 2245468.64)	1205.7 (747.74 to 1803.68)	0.03 (0 to 0.07)
Western Sub-Saharan Africa	763535.98 (488945.67 to 1106495.8)	1190.98 (751.89 to 1747.61)	2004403.78 (1280418.78 to 2908709.63)	1186.83 (745.4 to 1747.92)	−0.01 (−0.01 to −0.01)

AAPC, Average annual percent change; ASR, Age-standardized rate; CI, Confidence Intervals; UI, Uncertainty Intervals; SDI, Socio-Demographic Index.

### Stratification by SDI regions revealed critical disparities

In 2021, low-middle SDI regions bore the highest absolute burden (incidence: 1166.28/100,000 [748.76–1688.65]; prevalence: 15090.72 [11783.46–19122.42]; DALYs: 563.01 [67.78–1306.56]). However, the most rapid growth occurred in middle SDI regions, with AAPCs for incidence (0.17, 0.12–0.23), prevalence (0.23, 0.19–0.27), and DALYs (0.22, 0.18–0.26) surpassing other SDI categories. Similar accelerations were observed in upper-middle SDI regions (incidence AAPC: 0.14; prevalence: 0.15; DALYs: 0.13), contrasting with stagnations in high SDI areas.

### Geographic heterogeneity was evident across GBD regions

While most regions exhibited consistent growth, exceptions included Southern Sub-Saharan Africa, Western Sub-Saharan Africa, and High-Income North America, where declines occurred. Notably, East Asia demonstrated the steepest increases in incidence (AAPC: 0.26, 0.17–0.34), prevalence (0.29, 0.20–0.38), and DALYs (0.26, 0.18–0.34), followed by Andean Latin America and High-Income Asia Pacific. These patterns underscore how socioeconomic development and regional healthcare capacities differentially modulate migraine burden trajectories.

### Countries level

**Figure 2:**
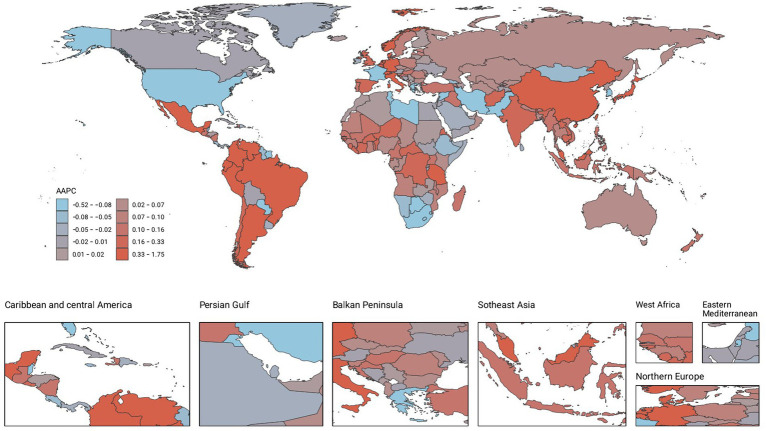
AAPC of DALYs.

**Figure 3:**
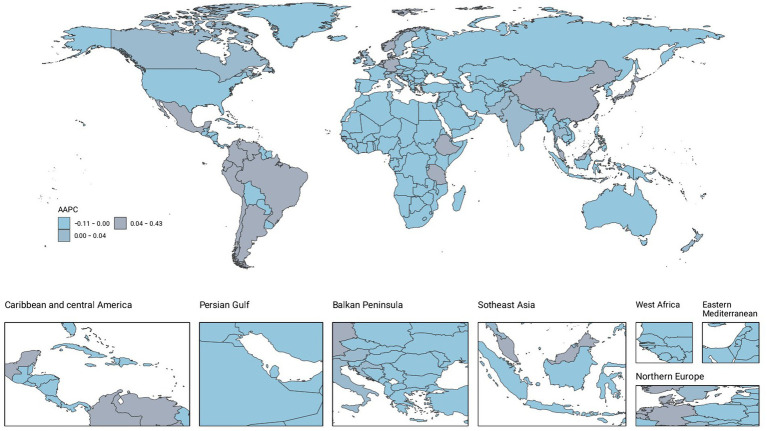
AAPC of Incidence.

**Figure 4:**
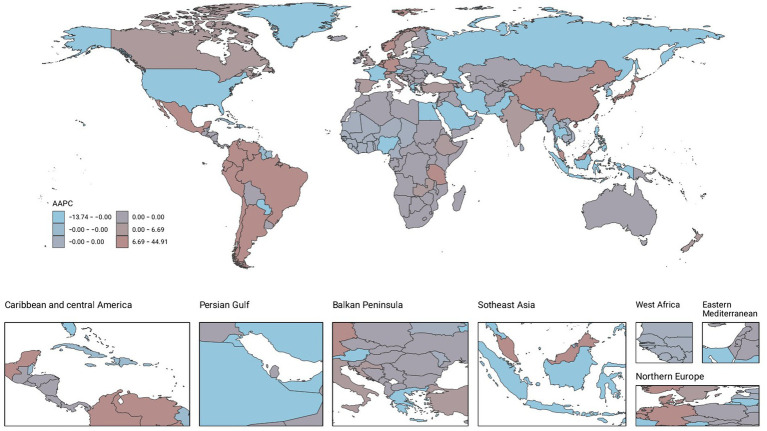
AAPC of Prevalence.

From 1990 to 2021, the global landscape of migraine among males aged 10–59 years showed significant disparities across countries. Among 204 nations, Belgium recorded the highest prevalence and DALYs, with 18970.49 cases per 100,000 people (95% UI: 24793.7 to 14277.75) and 691.32 (95% UI: 1636.39 to 65.12), respectively. Indonesia and the Philippines had the highest incidence, each with 1336.41 cases per 100,000 people (95% UI: 1932.13–861.81). From 1990 to 2021, Singapore experienced the most pronounced increases in prevalence, incidence, and DALYs, with AAPCs of 0.51 (95% CI: 0.51–0.51), 0.42 (95% CI: 0.42–0.42), and 0.4 (95% CI: 0.37–0.44), respectively. In contrast, the Republic of Korea showed the most significant decreases in incidence, prevalence, and DALYs, with AAPCs of −0.12 (95% CI: −0.21–0.03), −0.08 (95% CI: −0.15–0.02), and −0.09 (95% CI: −0.18--0), respectively (Figures 2–4; Supplementary Tables S3–S5).

### Joinpoint regression analysis

**Figure 5:**
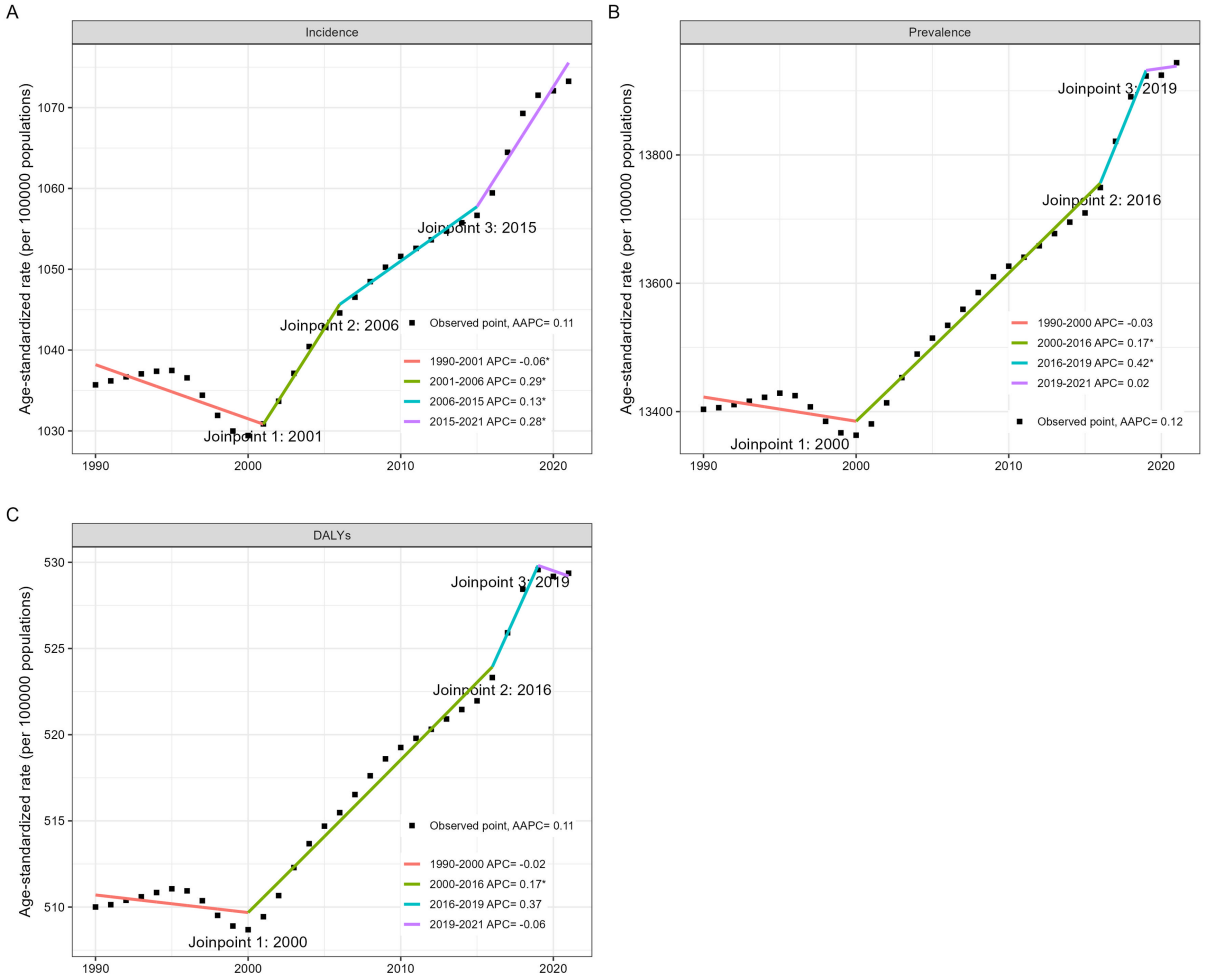
Joinpoint regression analysis for the global incidence **(A)**, prevalence **(B)**, DALYs **(C)** of migraine among males aged 10–59 years from 1990 to 2021.

Overall, from 1990 to 2021, the incidence, prevalence, and DALYs showed an upward trend (Table 2; Figure 5). The incidence had an AAPC of 0.11 (95% CI: 0.08–0.14) over the entire period, with a decline from 1990 to 2001, followed by a general increase from 2002 to 2021. The prevalence had an AAPC of 0.12 (95% CI: 0.08–0.16) throughout the study period, with a decrease from 1990 to 2000 but an increasing trend from 2001 to 2021. The most significant increase occurred between 2016 and 2019, with an annual percent change (APC) of 0.42 (95% CI: 0.06–0.79), although this trend flattened from 2019 to 2021, with an APC of 0.02 (95% CI: −0.34–0.39). DALYs had an AAPC of 0.11 (95% CI: 0.07–0.16) over the entire period, with declines from 1990 to 2000 and 2020 to 2021 but an overall increase from 2001 to 2019.

**Table 2:** The prevalence, incidence and DALYs of AAPCs in global and SDI regions.

	Incidence		Prevalence		DALYs	
	Period	APC	Period	APC	Period	APC
Global	1990–2021 (AAPC)	0.11 (0.08 to 0.14)	1990–2021 (AAPC)	0.12 (0.08 to 0.16)	1990–2021 (AAPC)	0.11 (0.07 to 0.16)
	1990–2001	−0.06 (−0.1 to −0.03)	1990–2000	−0.03 (−0.06 to 0)	1990–2000	−0.02 (−0.05 to 0.01)
	2001–2006	0.29 (0.14 to 0.43)	2000–2016	0.17 (0.16 to 0.19)	2000–2016	0.17 (0.16 to 0.19)
	2006–2015	0.13 (0.08 to 0.18)	2016–2019	0.42 (0.06 to 0.79)	2016–2019	0.37 (−0.03 to 0.78)
	2015–2021	0.28 (0.2 to 0.36)	2019–2021	0.02 (−0.34 to 0.39)	2019–2021	−0.06 (−0.46 to 0.35)
High SDI	1990–2021 (AAPC)	0.09 (0.08 to 0.11)	1990–2021 (AAPC)	0.07 (0.04 to 0.1)	1990–2021 (AAPC)	0.07 (0.03 to 0.1)
	1990–2005	−0.1 (−0.11 to −0.09)	1990–2005	−0.15 (−0.16 to −0.14)	1990–2003	−0.13 (−0.15 to −0.12)
	2005–2011	0.27 (0.24 to 0.31)	2005–2011	0.34 (0.27 to 0.41)	2003–2006	0.07 (−0.25 to 0.4)
	2011–2014	0.63 (0.46 to 0.8)	2011–2014	0.6 (0.29 to 0.9)	2006–2015	0.38 (0.35 to 0.42)
	2014–2021	0.13 (0.11 to 0.15)	2014–2021	0.09 (0.05 to 0.13)	2015–2021	0.01 (−0.04 to 0.07)
High-middle SDI	1990–2021 (AAPC)	0.14 (0.1 to 0.18)	1990–2021 (AAPC)	0.15 (0.1 to 0.19)	1990–2021 (AAPC)	0.13 (0.09 to 0.17)
	1990–2001	−0.09 (−0.13 to −0.06)	1990–2001	−0.06 (−0.1 to −0.02)	1990–2001	−0.08 (−0.12 to −0.05)
	2001–2005	0.64 (0.37 to 0.91)	2001–2005	0.51 (0.24 to 0.79)	2001–2005	0.51 (0.24 to 0.78)
	2005–2014	0.13 (0.07 to 0.18)	2005–2014	0.11 (0.05 to 0.17)	2005–2014	0.12 (0.06 to 0.18)
	2014–2021	0.26 (0.18 to 0.33)	2014–2021	0.32 (0.24 to 0.39)	2014–2021	0.28 (0.2 to 0.35)
Middle SDI	1990–2021 (AAPC)	0.17 (0.12 to 0.23)	1990–2021 (AAPC)	0.23 (0.19 to 0.27)	1990–2021 (AAPC)	0.22 (0.18 to 0.26)
	1990–1995	0.13 (−0.01 to 0.28)	1990–2001	0.09 (0.05 to 0.12)	1990–2001	0.09 (0.05 to 0.12)
	1995–2000	−0.2 (−0.4 to 0.01)	2001–2005	0.52 (0.26 to 0.79)	2001–2005	0.53 (0.25 to 0.8)
	2000–2005	0.5 (0.29 to 0.71)	2005–2015	0.18 (0.13 to 0.22)	2005–2015	0.17 (0.12 to 0.22)
	2005–2021	0.2 (0.18 to 0.23)	2015–2021	0.39 (0.3 to 0.48)	2015–2021	0.35 (0.26 to 0.44)
Low-middle SDI	1990–2021 (AAPC)	−0.01 (−0.03 to 0.01)	1990–2021 (AAPC)	0 (−0.03 to 0.03)	1990–2021 (AAPC)	0 (−0.04 to 0.03)
	1990–1995	−0.03 (−0.09 to 0.02)	1990–1996	−0.05 (−0.1 to 0)	1990–2001	−0.13 (−0.16 to −0.11)
	1995–2000	−0.3 (−0.38 to −0.22)	1996–1999	−0.38 (−0.66 to −0.09)	2001–2015	−0.01 (−0.03 to 0.01)
	2000–2015	−0.03 (−0.05 to −0.02)	1999–2015	−0.03 (−0.04 to −0.02)	2015–2019	0.4 (0.21 to 0.6)
	2015–2021	0.31 (0.26 to 0.35)	2015–2021	0.3 (0.26 to 0.35)	2019–2021	−0.09 (−0.48 to 0.3)
Low SDI	1990–2021 (AAPC)	−0.01 (−0.03 to 0.01)	1990–2021 (AAPC)	−0.01 (−0.03 to 0)	1990–2021 (AAPC)	0 (−0.03 to 0.04)
	1990–1996	−0.06 (−0.1 to −0.03)	1990–1996	−0.05 (−0.09 to −0.02)	1990–2003	−0.1 (−0.11 to −0.08)
	1999–1999	−0.25 (−0.44 to −0.05)	1999–2000	−0.23 (−0.34 to −0.12)	2003–2016	0.06 (0.04 to 0.08)
	1999–2015	0 (−0.01 to 0)	2000–2014	0 (−0.02 to 0.01)	2016–2019	0.26 (−0.04 to 0.56)
	2015–2021	0.13 (0.1 to 0.17)	2014–2021	0.13 (0.1 to 0.16)	2019–2021	−0.13 (−0.43 to 0.17)

### Age, period and cohort analysis

#### Age effects

The age-related impacts on incidence, prevalence, and DALYs are detailed in Table 3. The incidence rate ratio (RR) peaks in the 10–14 age group (RR = 1.798, 95% CI: 1.797–1.799) and declines with age. In contrast, prevalence and DALYs follow different patterns. The highest prevalence RR was observed in the 30–34 age group at 1.153 (95% CI: 1.153–1.153), while the 40–44 age group had the highest DALYs RR at 1.155 (95% CI: 1.154–1.155). Notably, the 30–34, 35–39, and 40–44 age groups presented similar burdens of prevalence and DALYs, indicating a sustained high disease burden within this age range. These findings suggest that while incidence is more common in younger populations, the disease burden accumulates and peaks in middle-aged individuals.

**Table 3:** Age, period, and cohort effects on the global burden of migraine in males from 1990 to 2021.

Factor	Incidence		Prevalence		DALYs	
	RR	95%CI	RR	95%CI	RR	95%CI
Age (years)						
10–14	1.798	1.797 to 1.799	0.690	0.690 to 0.690	0.659	0.659 to 0.660
15–19	1.221	1.220 to 1.222	0.983	0.983 to 0.983	0.972	0.971 to 0.973
20–24	1.126	1.125 to 1.126	1.080	1.079 to 1.080	1.066	1.065 to 1.066
25–29	1.158	1.158 to 1.159	1.128	1.128 to 1.128	1.097	1.096 to 1.097
30–34	1.101	1.100 to 1.101	1.153	1.153 to 1.153	1.132	1.131 to 1.133
35–39	1.124	1.123 to 1.124	1.149	1.149 to 1.150	1.152	1.151 to 1.153
40–44	0.971	0.970 to 0.971	1.132	1.132 to 1.132	1.155	1.154 to 1.155
45–49	0.760	0.759 to 0.760	1.034	1.034 to 1.034	1.067	1.066 to 1.068
50–54	0.702	0.702 to 0.703	0.938	0.938 to 0.939	0.970	0.969 to 0.971
55–59	0.546	0.545 to 0.546	0.831	0.831 to 0.832	0.856	0.855 to 0.857
Period						
1992–1996	1.051	1.050 to 1.051	0.974	0.974 to 0.975	0.969	0.968 to 0.969
1997–2001	1.022	1.022 to 1.022	0.978	0.978 to 0.978	0.975	0.975 to 0.976
2002–2006	1.006	1.006 to 1.006	0.991	0.991 to 0.992	0.991	0.990 to 0.991
2007–2011	0.990	0.989 to 0.990	1.005	1.005 to 1.005	1.007	1.006 to 1.007
2012–2016	0.971	0.971 to 0.972	1.016	1.016 to 1.016	1.019	1.019 to 1.020
2017–2021	0.963	0.963 to 0.964	1.037	1.037 to 1.037	1.040	1.040 to 1.041
Cohort						
1937–1941	0.845	0.843 to 0.847	1.051	1.050 to 1.051	1.072	1.069 to 1.074
1942–1946	0.865	0.863 to 0.866	1.039	1.039 to 1.039	1.054	1.052 to 1.056
1947–1951	0.883	0.881 to 0.884	1.027	1.027 to 1.028	1.039	1.038 to 1.040
1952–1956	0.908	0.907 to 0.909	1.019	1.019 to 1.020	1.029	1.028 to 1.030
1957–1961	0.935	0.934 to 0.936	1.018	1.018 to 1.018	1.023	1.022 to 1.024
1962–1966	0.957	0.956 to 0.957	1.010	1.010 to 1.011	1.012	1.011 to 1.013
1967–1971	0.975	0.974 to 0.976	0.996	0.996 to 0.997	0.996	0.995 to 0.997
1972–1976	1.00	0.999 to 1.000	0.991	0.991 to 0.991	0.989	0.988 to 0.990
1977–1981	1.028	1.028 to 1.029	0.993	0.993 to 0.993	0.989	0.988 to 0.989
1982–1986	1.049	1.049 to 1.050	0.990	0.990 to 0.990	0.984	0.984 to 0.985
1987–1991	1.067	1.067 to 1.068	0.978	0.977 to 0.978	0.971	0.971 to 0.972
1992–1996	1.098	1.098 to 1.099	0.979	0.979 to 0.979	0.970	0.970 to 0.971
1997–2001	1.135	1.135 to 1.136	0.984	0.983 to 0.984	0.973	0.972 to 0.974
2002–2006	1.162	1.161 to 1.163	0.975	0.975 to 0.975	0.964	0.962 to 0.965
2007–2011	1.174	1.173 to 1.175	0.955	0.955 to 0.956	0.945	0.943 to 0.946

#### Period effects

The period effects on incidence, prevalence, and DALYs are presented in Table 3. All three metrics display distinct temporal trends. The incidence decreases from 1992 to 1996 (RR = 1.051, 95% CI: 1.050–1.051) to 2017 to 2021 (RR = 0.963, 95% CI: 0.963–0.964). Conversely, prevalence and DALYs show an increasing trend, peaking from 2017 to 2021 (prevalence RR = 1.037, 95% CI: 1.037–1.037; DALYs RR = 1.040, 95% CI: 1.040–1.041). This difference may reflect improvements in disease management and survival rates over time.

#### Cohort effects

The cohort effects on incidence, prevalence, and DALYs are outlined in Table 3. Cohort analysis reveals intriguing generational patterns. The incidence was lowest in the earliest birth cohort (1937–1941: RR = 0.845, 95% CI: 0.843–0.847) and highest in the later cohort (2007–2011: RR = 1.174, 95% CI: 1.173–1.175). However, prevalence and DALYs show the opposite trend, with early cohorts experiencing higher rates (1937–1941 cohort: prevalence RR = 1.051, 95% CI: 1.050–1.051; DALYs RR = 1.072, 95% CI: 1.069–1.074), whereas later cohorts are lower.

### Decomposition analysis of migraine among males aged 10–59 years from 1990 to 2021 according to the SDI and 21 GBD regions

**Figure 6:**
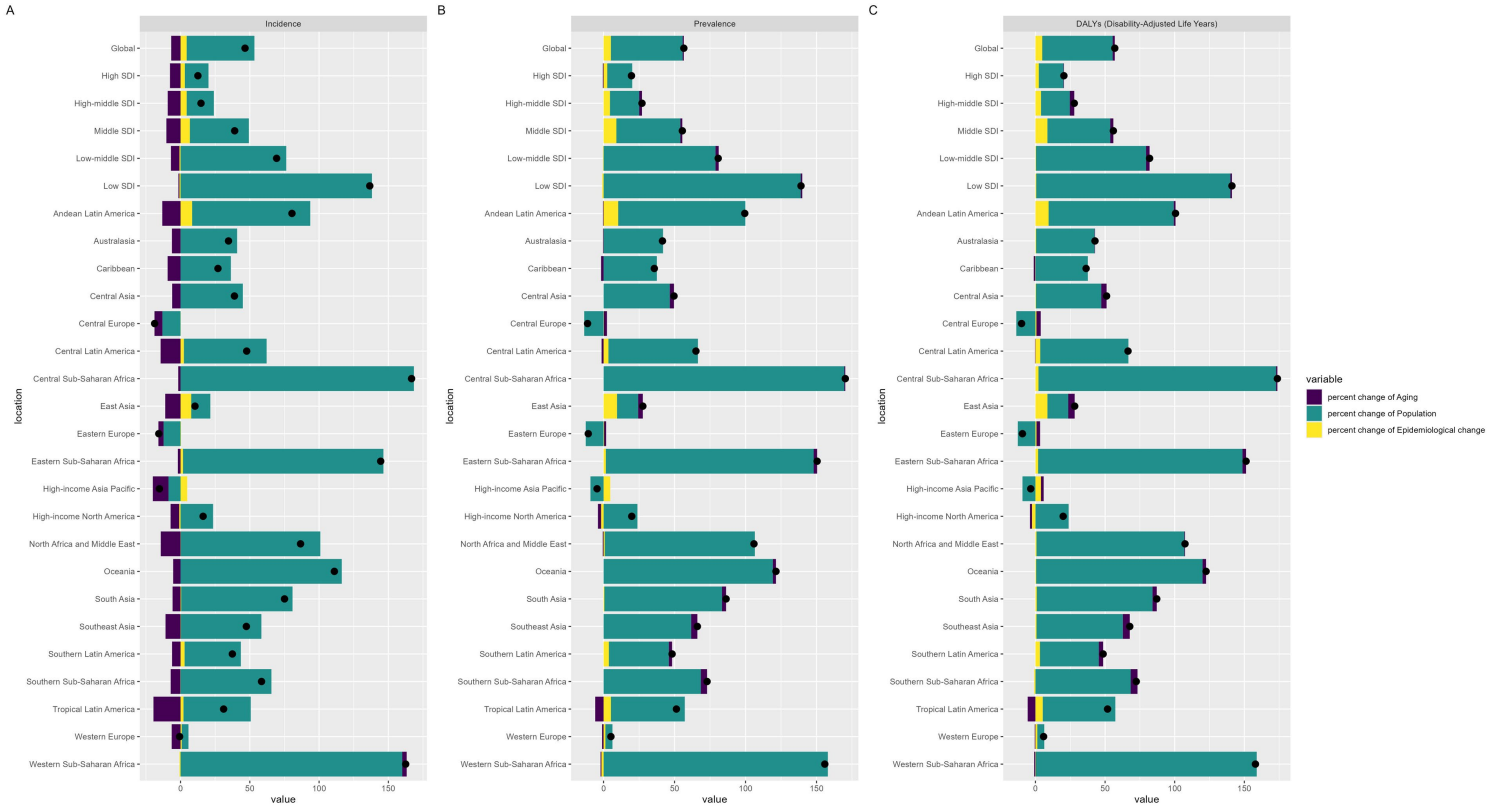
Decomposition analysis of migraine incidence, prevalence and DALYs according to the SDI and 21 GBD regions from 1990 to 2021.

Our decomposition analysis elucidates the relative contributions of aging, population growth, and epidemiological changes to the shifting incidence, prevalence, and DALYs of migraine among males aged 10–59 across five SDI regions and 21 GBD regions (Figure 6; Supplementary Table S6). Notably, from 1990 to 2021, population growth was the primary driver of the global disease burden. We observed that aging had a negative impact on migraine incidence in most countries. Additionally, in Central Europe, Eastern Europe, and the high-income Asia Pacific region, a reduction in disease burden was attributed mainly to decreased population growth. In regions experiencing population increases, this growth became the main driver of the increasing disease burden.

### Future burden of migraine

**Figure 7:**
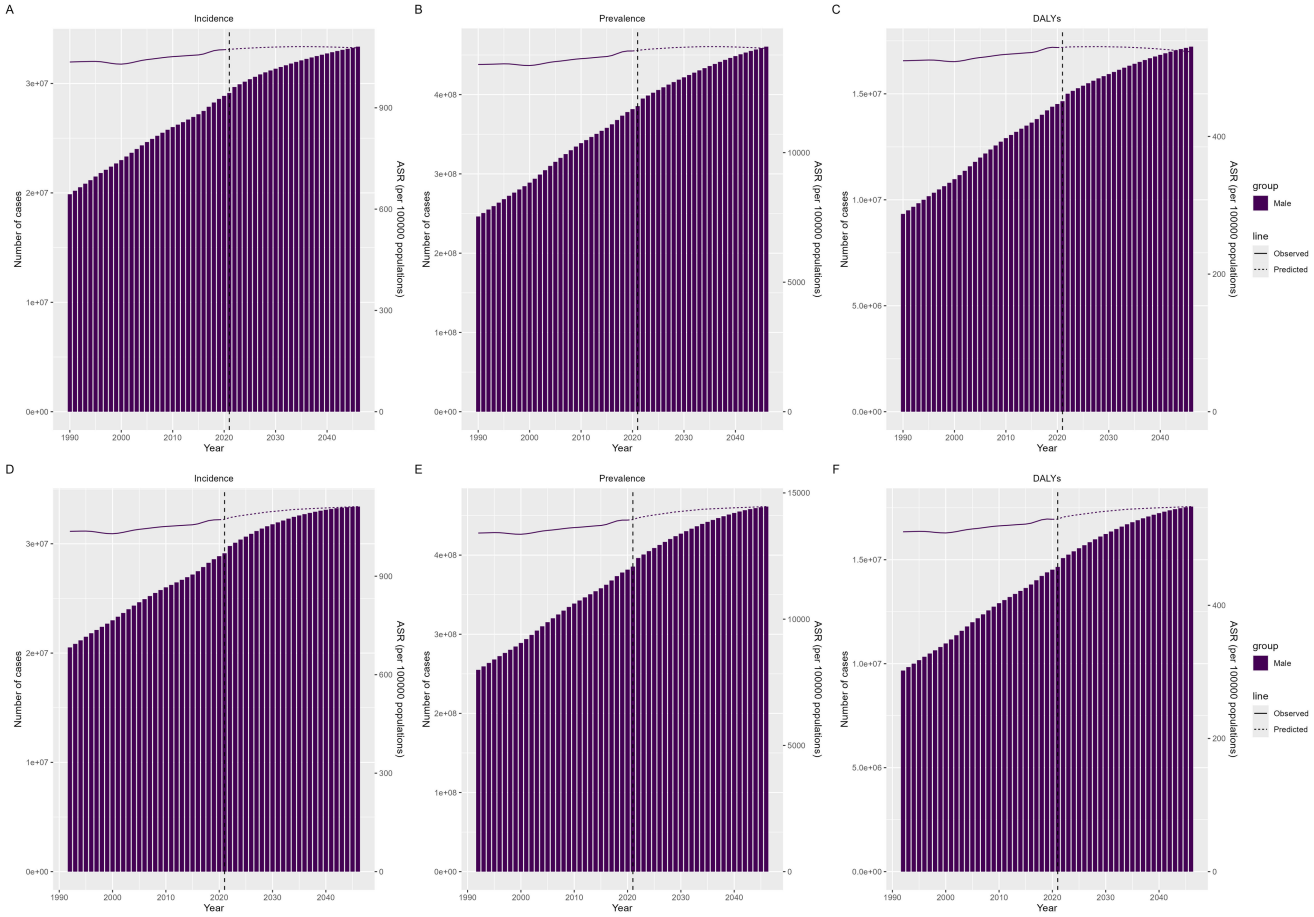
Future forecasts of the GBD in migraine incidence, prevalence and DALYs. **(A–C)** Predicted by BAPC, **(D–F)** Predicted by Nordpred.

Figure 7 illustrates the projected trends of migraine among males aged 10–59 years globally. Both of our predictive models indicate that the number of incident cases, prevalent cases, and DALYs will increase, with the ASR also showing an increasing trend. These findings suggest that the disease burden of migraine in males aged 10–59 years may further increase in the future.

## Discussion

This study provides an in-depth analysis of the epidemiological characteristics of migraine in men aged 10–59 years globally, revealing a unique pattern of burden of the disease in the male population. The findings show that although the overall burden of migraine in men is lower than in women, its internal heterogeneity cannot be ignored. This study utilized data from GBD 2021 to analyze the epidemiological characteristics of migraine among males aged 10–59 years globally from 1990 to 2021. Our main findings include the following: (1) the burden of migraine in this population has shown an increasing trend worldwide, with AAPCs in incidence, prevalence, and DALYs of 0.11 (95% CI: 0.08–0.14), 0.12 (95% CI: 0.08–0.16), and 0.11 (95% CI: 0.07–0.16), respectively (3). (2) The disease burden in high-, high-, middle-, and middle-SDI regions also showed a significant increasing trend, with the fastest growth occurring in the middle-SDI region, whose AAPCs in incidence, prevalence, and DALYs were 0.17 (95% CI: 0.12–0.23), 0.23 (95% CI: 0.19–0.27), and 0.22 (95% CI: 0.18–0.26), respectively. (3) Regional analysis revealed that the overall trend in this population is similar to that of the general population; however, in high-income Asia Pacific countries, the disease burden showed an increasing trend, differing from other related studies (15, 23), with AAPCs for incidence, prevalence, and DALYs of 0.14 (95% CI: 0.12–0.17), 0.14 (95% CI: 0.11–0.16), and 0.11 (95% CI: 0.09–0.13), respectively. (4) At the national level, we found that Singapore had the largest increase in disease burden, while the Republic of Korea showed the most significant decrease, both of which are high-SDI regions. (5) Using an age–period–cohort model, we explored the impact of different ages, periods, and cohorts on the migraine burden in this population. We found that the incidence was highest among patients aged 10–14 years, whereas the prevalence and DALYs peaked at ages 30–44 years. From 1992 to 2021, incidence showed a declining trend, whereas prevalence and DALYs increased. Newer birth cohorts presented higher incidence rates but lower prevalence rates and DALYs. (6) Decomposition analysis indicated that population growth is the main factor driving the increase in migraine burden. (7) Various predictive models consistently suggest that the migraine burden among males aged 10–59 will continue to rise in the coming decades. This finding suggests that the burden of migraine in men is not uniformly distributed, but that there are significant age-stage differences. Adolescence as the peak incidence may be closely related to the physiological, psychological, and social stresses that characterize that stage; whereas the peak of DALYs at 30–34 years of age may reflect a combination of occupational stresses, family responsibilities, and other multiple factors.

In addition, this study found significant regional differences in the burden of migraine in men. Stratification by socio-demographic index (SDI) showed that the growth rate of DALYs slowed down after age 50 years for men in high SDI countries (APC = 0.8%), whereas it continued to rise in low and medium SDI countries (APC = 2.1%), suggesting a moderating effect of healthcare resource accessibility on the burden of older men. This difference may stem from disparities in the allocation of healthcare resources and the level of disease prevention and treatment in different SDI countries. High SDI countries usually have better healthcare systems and higher levels of disease awareness, and are able to effectively control the progression and complications of migraine, whereas low and middle SDI countries may have limited healthcare resources and insufficient disease awareness, which may result in the burden of migraine continuing to increase in old age. This intra-gender heterogeneity challenges the simplistic conclusion that men have a lower burden than women.

The upward trend in global incidence, prevalence and DALYs we observed aligns with previous research findings (24, 25), indicating that the disease burden of migraine continues to exhibit a persistent growth pattern worldwide. However, our study notably reveals that certain regions bear the brunt of this escalating burden, including Andean Latin America, Central Latin America, Southern Latin America, Tropical Latin America, East Asia, high-income Asia Pacific, and Western Europe. Concurrently, we observed a rapid increase in the disease burden of migraine in high-SDI countries such as Singapore and Japan. The sharp increase in migraine burden in regions such as Latin America, East Asia, and high-income Asia Pacific may be associated with the rapid pace of urbanization and economic development, as well as improvements in local disease diagnosis and treatment standards (26). As these regions undergo significant changes in living conditions, work environments, and social structures, individuals may experience increased stress levels and lifestyle alterations, which are recognized risk factors for migraine (27–30). Studies indicate that living in urban environments induces functional neurological changes, increasing activity in brain regions associated with stress processing. This phenomenon elucidates why urban dwellers are more susceptible to headaches, given that stress is a common trigger for all types of headaches (31). Furthermore, in many countries across Latin America and East Asia, inadequate healthcare standards and improper medication management contribute to the exacerbation of the disease burden (32). Our study revealed that low-middle-SDI regions presented the highest prevalence, incidence, and DALYs, while high-, high-middle-, and middle-SDI regions demonstrated a rapidly increasing trend in migraine disease burden among their populations. These findings align with previous research findings (15). Our research indicates that middle-SDI regions experience the most rapid growth in disease burden. The most plausible hypothesis for this outcome is that economic development in these areas has led to an increase in migraine triggers and improved disease diagnosis reporting (26, 33). Moreover, rapid urbanization and industrialization have accelerated changes in people’s lifestyles, encompassing in sedentary habits, heightened stress levels, a lack of physical exercise, excessive use of medications and electronic devices, and worsening sleep patterns, all of which exacerbate the occurrence of migraines (27, 34–36).

Our study revealed that from 1990 to 2021, the migraine burden among males aged 10–59 years in high-income Asia–Pacific regions showed an increasing trend, contrary to the findings of previous studies (15, 23). This discrepancy may be due to a rise in healthcare-seeking behavior among men, growing public awareness of migraines, and external pressures (37, 38). Earlier research indicated that women generally have a lower pain threshold than men do (39, 40), leading to higher healthcare demand (37). However, as the understanding of migraine improves, stigma decreases, resulting in increased diagnosis rates among men (38). Additionally, rapid economic growth in high-income Asia–Pacific regions has increased social, work, and family pressures on men, as well as unhealthy behaviors, contributing to the increasing migraine burden (41, 42). Nonetheless, there is a lack of research specifically on male migraines, limiting our ability to analyze the reasons behind this trend. Future studies should focus more on male migraines to explore these factors in depth.

In our research, we found that adolescents have a higher incidence rate, whereas middle-aged and elderly individuals have a higher prevalence rate and DALYs. We observed that the age effect analysis revealed a peak in migraine incidence during adolescence (10–14 years old), followed by a decline with increasing age. This finding aligns with those of previous studies, which indicate that migraines typically onset during puberty (36, 43, 44). However, prevalence and DALYs exhibit a different pattern, peaking in the 30–44 age group and maintaining high levels thereafter. This discrepancy may reflect the cumulative effects and chronification process of migraine, which is consistent with the findings of Scher et al. (45) on headache chronification. Additionally, it is worth considering whether there are causal links beyond the previously established positive correlation (46) between the increasingly prominent burden of adolescent migraines and the increasing incidence of school bullying in recent years. The period effect analysis reveals an intriguing trend: despite a gradual decline in incidence over time, both the prevalence and DALYs show an upward trajectory. This seemingly paradoxical phenomenon may reflect improvements in disease management and increased patient survival rates, which is consistent with the findings of Steiner et al. (47) on the global burden of headache. Furthermore, this may indicate that while new cases are decreasing, the prolonged course of illness or worsening symptoms among existing patients leads to an overall increase in disease burden. The cohort effect analysis demonstrated that later-born cohorts presented higher incidence rates but lower prevalence rates and DALYs. This generational disparity reflects the influence of multiple factors, including changes in diagnostic criteria, improved health awareness, and intergenerational lifestyle differences (26, 33). For example, younger cohorts may have easier access to accurate diagnoses while potentially benefiting from improved treatment methods, thereby reducing long-term disease burden.

To alleviate the disease burden of migraine, a multifaceted approach is essential. Unlike other non-communicable diseases, such as stroke, epilepsy, and cancer, migraine does not result in mortality and can resolve spontaneously. This has led to insufficient medical attention given to migraine. Therefore, our aim is to implement public health initiatives that enhance awareness about migraine, its triggers, and management strategies, thereby enabling individuals to better comprehend and cope with this condition (48, 49). Moreover, given the diverse stressors and migraine triggers experienced by different age groups, it is imperative to adopt tailored therapeutic approaches for various migraine patients, striving to achieve optimal treatment outcomes (48, 50, 51). Our study also revealed that adolescents have a greater incidence of migraines, while the prevalence and DALYs are greater in middle-aged and older adults. Based on the basis of these results, we should focus on primary prevention in adolescents by providing psychological support, encouraging healthy lifestyle habits (52), and increasing awareness and understanding of migraines (53). For middle-aged and older groups, we should aim to prevent migraine recurrence through enhanced secondary prevention measures, such as the development of medications (36, 54), the promotion of cognitive–behavioral therapy (55), mindful dietary intake (56), and improving patients’ knowledge and self-management skills.

### Limitations

This study has several limitations. The data obtained from the GBD largely rely on modeling, as GBD collaborators employ numerous statistical modeling methods, particularly in countries where original data are scarce. Furthermore, most population-level studies have been conducted in developed countries, with limited data from developing nations. Therefore, it is crucial to encourage global epidemiological research on migraine to reduce data scarcity, especially in developing countries, and to make our model estimates more reliable. Additionally, owing to the lag in GBD data, it is essential to integrate real-world data to provide a more accurate and comprehensive assessment of the migraine burden. The lack of research on migraines in males has resulted in insufficient data on related risk factors. To explore these factors further, studies should incorporate real-world data and develop targeted prevention strategies.

## Conclusion

Between 1990 and 2021, the global burden of migraine among individuals aged 10–59 years increased substantially, revealing notable disparities across SDI regions, countries, age groups, and sexes. In 2021, middle-SDI regions reported the highest absolute numbers of prevalent cases, incident cases, and DALYs, whereas low-middle-SDI regions presented the highest incidence rates, prevalence rates, and DALYs. Globally, incidence rates peaked among patients aged 10–14 years, whereas prevalence rates and DALYs reached their zenith in the 30–44 years age group. From 1992 to 2021, incidence rates demonstrated a declining trend, whereas prevalence rates and DALYs exhibited an upward trajectory. More recent birth cohorts presented higher incidence rates but lower prevalence rates and DALYs. Factors such as rapid urbanization, economic development, and high-stress lifestyles may contribute to the increased migraine burden in certain regions. This necessitates targeted interventions and public health strategies, particularly in severely affected areas. Future research should prioritize identifying risk factors and enhancing diagnostic and treatment strategies. Prevention measures should be tailored to different groups: implement primary prevention for adolescents (high incidence group) and actively pursue secondary prevention for middle-aged and older adults (high prevalence and DALYs group) while optimizing tertiary prevention strategies.

## Data Availability

The GBD study 2021 data resources were available online from the Global Health Data Exchange (GHDx) query tool (http://ghdx.healthdata.org/gbd-results-tool).

## Footnotes

^1^ http://ghdx.healthdata.org/gbd-results-tool
